# A Systematic Review and Meta-Analysis of the Haemodynamic Effects of Cannabidiol

**DOI:** 10.3389/fphar.2017.00081

**Published:** 2017-02-24

**Authors:** Salahaden R. Sultan, Sophie A. Millar, Timothy J. England, Saoirse E. O'Sullivan

**Affiliations:** Vascular Medicine, Division of Medical Sciences and Graduate Entry Medicine, Royal Derby Hospital Centre, School of Medicine, University of NottinghamDerby, UK

**Keywords:** cannabidiol, Epidiolex, CBD, cardiovascular system, haemodynamic, blood pressure, heart rate, blood flow

## Abstract

Despite cannabidiol (CBD) having numerous cardiovascular effects *in vitro*, its haemodynamic effects *in vivo* are unclear. Nonetheless, the clinical use of CBD (Epidiolex) is becoming more widespread. The aim of this systematic review was to establish whether CBD is associated with changes in haemodynamics *in vivo*. Twenty-five studies that assessed the haemodynamic effects of CBD (from PubMed, Medline and EMBASE) were systematically reviewed and meta-analyzed. Data on blood pressure (BP), heart rate (HR), and blood flow (BF) were extracted and analyzed using random effects models. Twenty-two publications assessed BP and HR among 6 species (BP *n* = 344 and HR *n* = 395), and 5 publications assessed BF in 3 species (*n* = 56) after acute dosing of CBD. Chronic dosing was assessed in 4 publications in 3 species (total subjects BP, *n* = 6; HR, *n* = 27; BF, *n* = 3). Acute CBD dosing had no effect on BP or HR under control conditions. Similarly, chronic dosing with CBD had no effect on HR. In models of stress, acute CBD administration significantly reduced the increase in BP and HR induced by stress (BP, mean difference (MD) −3.54, 95% CI −5.19, −1.9, *p* < 0.0001; HR, MD −16.23, 95% CI −26.44, −6.02, *p* = 0.002). In mouse models of stroke, CBD significantly increased cerebral blood flow (CBF, standardized mean difference (SMD) 1.62, 95% CI 0.41, 2.83, *p* = 0.009). Heterogeneity among the studies was present, there was no publication bias except in HR of control and stressful conditions after acute CBD dosing, and median study quality was 5 out of 9 (ranging from 1 to 8). From the limited data available, we conclude that acute and chronic administration of CBD had no effect on BP or HR under control conditions, but reduces BP and HR in stressful conditions, and increases cerebral blood flow (CBF) in mouse models of stroke. Further studies are required to fully understand the potential haemodynamic effects of CBD in humans under normal and pathological conditions.

## Introduction

Cannabidiol (CBD) is the second most abundant phytocannabinoid, after Δ^9^-tetrahydrocannabinol (THC) (Pertwee, 2006; Tambaro and Bortolato, 2012) and was first isolated from the cannabis extract in 1940 (Adams et al., 1940). The pharmacological actions of CBD are complex; CBD has a low affinity to the cannabinoid receptor 1 (CB_1_) and cannabinoid receptor 2 (CB_2_) (Thomas et al., 2007; Pertwee, 2008), and targets a wide range of other receptors including 5-hydroxytryptamine (5-HT_1a_), transient receptor potential vanilloid receptor 1 (TRPV1), peroxisome proliferator-activated receptors (PPARs) and G protein-coupled receptor 55 (GPR55) (Pertwee, 2008; Stanley et al., 2013a). It is suggested that CBD may have therapeutic effects in a variety of disorders including diabetes, gastrointestinal disturbances, cancer, oxidative stress, inflammation and in cardiovascular disease (Russo and Guy, 2006; Capasso et al., 2008; Zuardi, 2008; Iuvone et al., 2009; Booz, 2011). CBD may also have desirable effects in multiple neurological and psychological disorders, including dystonia, schizophrenia, epilepsy and Parkinson's disease (Consroe et al., 1986; Leweke et al., 2012; Chagas et al., 2014; Devinsky et al., 2016). In a recent open-label trial investigating the effects of CBD (Epidiolex, 2–50 mg/kg) on 214 young patients with treatment-resistant epilepsy, CBD reduced seizure frequency and demonstrated an acceptable safety profile (Devinsky et al., 2016). Epidiolex was also safe and well tolerated in refractory epilepsy secondary to tuberous sclerosis and for epileptic spasms, highlighting its potential as a new treatment for refractory epilepsy (Abati et al., 2015; Geffrey et al., 2015).

Pre-clinical studies on the effects of CBD on the cardiovascular system have shown that CBD causes endothelium- and nitric oxide-dependent vasorelaxation of isolated human mesenteric arteries (Stanley et al., 2015) and PPARγ-dependent vasorelaxation of the rat aorta (O'sullivan et al., 2009). CBD also improves vasorelaxation in the femoral arteries of Zucker diabetic fatty rats via enhanced production of vasodilator COX-1/2-derived products acting at EP4 receptors (Stanley et al., 2013b; Wheal et al., 2014). CBD also decreases myocardial infract size in a rat model of ischaemia/reperfusion injury (Durst et al., 2007), attenuates myocardial dysfunction and inflammation in an animal model of diabetes (Rajesh et al., 2007), and attenuates inflammatory and oxidative stress changes induced by high glucose in human coronary artery cells (Rajesh et al., 2010). CBD also reduces cerebral vascular inflammation and associated dilatation induced by lipopolysaccharide in mice (Ruiz-Valdepenas et al., 2011), infarct size in animal models of stroke (England et al., 2015) and reduced blood brain barrier permeability (Hind et al., 2016). These effects on the cerebral vasculature appear to involve 5HT_1A_ and PPARγ. Together, these pre-clinical studies might suggest that the cardiovascular system is a therapeutic target for CBD (Stanley et al., 2013a). However, despite these many vascular effects of CBD, it is not yet clear whether CBD administration alters haemodynamics under control or pathological situations.

Given the increasing clinical use of CBD, and the numerous effects of CBD in the cardiovascular system, the aim of the present study was to systematically review and analyse *in vivo* studies evaluating the effects of CBD on alterations in haemodynamics.

## Materials and methods

### Search strategy

All studies potentially investigating the haemodynamic effect of CBD (including BP, HR, and BF) were searched (until November 2016) in Medline, EMBASE, and PubMed. Search keywords included: Cannabidiol, Epidiolex, cardiovascular, blood pressure (BP), systolic, diastolic, hypertension, hypotension, heart rate (HR), tachycardia, bradycardia, blood flow (BF), haemodynamic, vasodilatation, vasorelaxation, and vasoconstriction. References from included studies were also hand searched. Initially, the National Institute for Health and Excellent Care platform was used in which two databases (EMBASE and Medline) were used for searching. Then, a separate search was conducted using PubMed. Pre-specified inclusion and exclusion criteria were used to prevent bias; studies had to be *in vivo*, assess haemodynamics (BP, HR, or BF), be original articles, and be a controlled study. The exclusion criteria were: *in vitro* studies, mixtures of CBD with other cannabis extracts, studies not assessing haemodynamics (BP, HR, or BF), review articles and editorials, or uncontrolled studies.

### Data acquisition

Data on BP, HR, and BF were extracted from the included papers, and the changes in haemodynamics at 2 h post-drug after acute CBD dosing were used for analysis. A standardized time point of 2 h was decided as this was commonly available throughout the articles and CBD has been previously shown to peak at 2 h in plasma (Nadulski et al., 2005a,b). If there were no measurements taken at this time point (2 h post-drug) the closest time point to 2 h was used for analysis. In chronic studies, the mean of total measurements or measurements taken at the end of the study were used for analysis depending on data provided. If the exact number of animals used in each drug group were not available, the authors were contacted. If the authors were not able to provide the necessary information, the lowest number of animals within the range given was used for the experimental group CBD, and the highest number was used for the control group. If a crossover design was used in a study, the total number of humans was distributed equally to the drug groups. Grab application (version 1.5) was used to extract values from figures given in published articles if no values were stated within the text. If published articles used multiple groups (e.g., to assess dose-dependent effects) with one control group, then the number of humans or animals per control group was divided into the number of comparison groups. For the dose-response analysis, the total dose of the drug administrated to species up to the time in which the haemodynamics were measured was used.

### Quality

The methodological quality was assessed to identify risk of bias using six-point criteria derived from the Cochrane collaborations tool for assessing risk of bias (Higgins et al., 2011) and Stroke Therapy Academic Industry Recommendations (STAIR) (Stroke Therapy Academic Industry Roundtable, 1999). Each of the following criteria was equal to 1 point: randomisation, allocation concealment, blinding of outcome assessment, blinding of personnel and participant, assessment of more than one outcome, dose-response relationship, therapeutic time window, assessment of outcome >24 h and incomplete outcome data.

### Data analysis

Studies were divided into two groups (i.e., acute and chronic). Data were grouped before analysis according to model (non-stress and stress), and then sub-grouped by species (human, mice, rats, etc.). For the CBD dose-response analysis, data were grouped according to endpoint (BP, HR, or BF), and then sub-grouped according to dose. Data from each group were analyzed as forest plots using the Cochrane Review Manager software (Version 5.3. Copenhagen: The Nordic Cochrane Centre, The Cochrane Collaboration, 2014), and as funnel plots using Stata (StataCorp. 2009. Stata Statistical Software: Release 11. College Station, TX, USA). Funnel plot asymmetry (publication bias) was tested by Egger's test (Egger et al., 1997). Stata was also used for meta-regression that described the relationship between CBD dose and effect size. PRISM 7 (GraphPad, Software, La Jolla, CA, USA) was used to produce figures of dose-response. Since heterogeneity was expected between study protocols (different species, models, dose and time) random-effect models were used. The results of continuous data on BP and HR are expressed as mean difference (MD), and as standardized mean difference on BF with 95% confidence intervals (CIs) due to the different scales used in assessing BF. Studies were weighted by sample size and statistical significance was set at *p* < 0.05.

## Results

**Figure 1:**
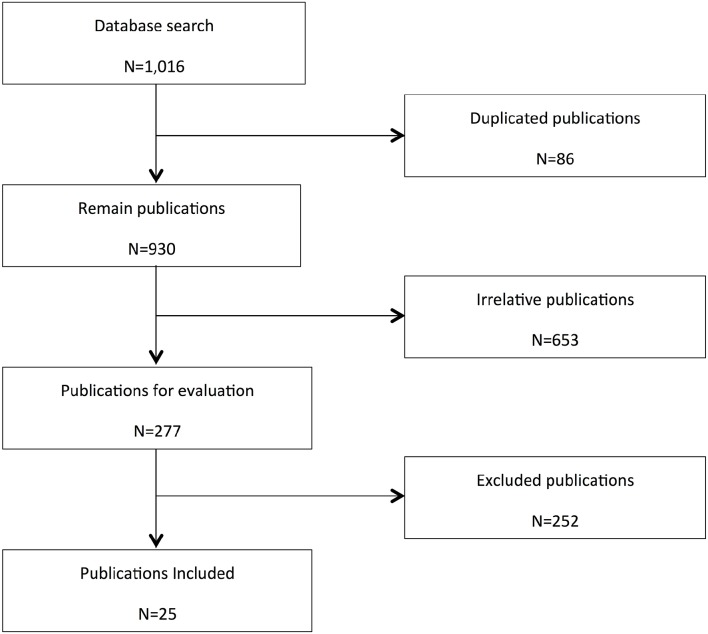
**Flow chart for study retrieval and selection**. *In vitro* studies, interaction studies including mixtures of CBD with other cannabis extracts, studies not assessing haemodynamics (BP, HR, or BF), review articles and editorials, or uncontrolled studies were excluded.

From the initial 1016 search results, 277 relevant publications were identified from three databases (Medline, EMBASE, and PubMed). Of these, 25 articles met the inclusion criteria (see Figure 1). A summary of the data extracted from included studies is shown in Table 1.

**Table 1:** **Summary of included studies in chronological order**.

**Reference**	**Species**	**Model**	**Dose (CBD)**	**Route**	**Time of CBD administration**	**Time of haemodynamic measurements**	**Finding**
Borgen and Davis, 1974	Rabbits	Anaesthetised	25 mg/kg	i.v.	Pre-test	Pre-drug and hourly interval to 7 h post-drug	No changes on HR
Bright et al., 1975	Dogs	Anaesthetised	0.5 or 1 mg/kg	i.v.	Pre-test	During 30 min post-drug.	↑MBP and HR
Belgrave et al., 1979	Humans	Healthy volunteers	320 μg/kg	Oral	Pre-test	Pre-drug and at 1.5, 2.5 and 3.5 h post-drug	No changes on HR
Gong et al., 1984	Human	Healthy volunteers	100, 600 or 1200 mg	Oral	Pre-test	Hourly interval to 6 h post-dru	No changes on SBP, DBP or HR
			1200 mg		Pre-test	Hourly interval to 6 h post-drug	
			1200 mg		Single dose per day for 20 days	Hourly interval to 6 h post-drug and on days 5, 12 and 19	
Consroe et al., 1991	Human	Patients with Huntington disease	10 mg/kg	Oral	Single dose per day for 6 weeks	Pre-drug, during and post-drug	No changes on MBP or HR
Zuardi et al., 1993	Human	Healthy volunteers	300 mg	Oral	Pre-test	Pre-drug and Post drug at 80 min (pre-stress), 85 min (during stress) and 100 min (post-stress)	No changes on SBP or HR
Mishima et al., 2005	Mice	MCAO	3 mg/kg	i.p.	Pre and 3 h post-occlusion	MBP and HR: 2 h post-occlusion	No changes on MBP or HR
						CBF: during 4 h post-occlusion	↑CBF
Resstel et al., 2006	Rats	Stress (fear)	10 mg/kg	i.p.	Pre-test	Pre-stress and during 10 min post-stress	↓MBP and HR post-stress
Hayakawa et al., 2007a	Mice	MCAO	3 mg/kg	i.p.	Pre, 3 and 4 h post-occlusion, and 1 and 2 h post-reperfusion	MBP and HR: pre-reperfusio	No changes on MBP or HR
						CBF: during 4 h of occlusion and post-reperfusion	↑CBF
Hayakawa et al., 2007b	Mice	MCAO	3 mg/kg	i.p.	Pre-occlusion and 3 h post-occlusion and Single dose per day for 14 days	During 4 h and on day 14 post-occlusion	↑CBF
Durst et al., 2007	Rats	Myocardial infarction	5 mg/kg for 7 days	i.p.	Pre-ischaemia and post-ischaemia for 7 days	On day 1 and 7 post-ischaemia	No changes on HR
Alvarez et al., 2008	Piglets	Carotid occlusion and hypoxic ischaemia	0.1 mg/kg	i.v.	15 min and 4 h post-procedure	MBP and HR: pre-procedure and at 3 and 6 h post-procedure	Maintain MBP and HR after the fall post- hypoxic ischaemia
						CBF: pre-procedure and during 6 h post-procedure	↑CBF
Hayakawa et al., 2008	Mice	MCAO	3 mg/kg	i.p.	Pre-occlusion and 3 h post-occlusion	During 4 h post-occlusion	No changes on MBP or HR
Resstel et al., 2009	Rats	Stress (restraint)	1, 10, or 20 mg/kg	i.p.	Pre-stress	Pre-stress and during 1 hr post-stress	↓MBP and HR post-stress
Alves et al., 2010	Rats	Conscious	60 nmol	BNST	Post-ACSF	During 60 min post-drug	No changes on MBP or HR
Walsh et al., 2010	Rats	Myocardial infarction	10 or 50 μg/kg	i.v.	Pre-ischaemia and pre-reperfusion	Pre- ischaemia during 2.30 h post-ischaemia,	↓MBP
Granjeiro et al., 2011	Rats	Stress (restraint)	15, 30 or 60 nmol	intracisternal	Pre-stress	Pre-stress and during 1 hr post-stress	No change in MBP or HR
Hallak et al., 2011	Human	Healthy volunteers	600 mg	Oral	Pre-test	Pre-drug and at 30 min interval for 2.30 h post-drug	No change in SBP, DBP or HR
Gomes et al., 2012	Rats	Stress (fear)	15, 30 or 60 nmol	BNST	Pre-stress	Pre-stress and during 10 min post-stress	30 and 60 nmol: ↓MBP and HR
Martin-Santos et al., 2012	Human	Healthy volunteers	600 mg	Oral	Pre-test	Pre-drug and Hourly interval for 3 h post-drug	No changes on BP or HR
Gomes et al., 2013	Rats	Stress (restraint)	15, 30 or 60 nmol	BNST	Pre-stress	Pre-stress and during 60 min post-stress	Enhanced the HR increase post-stress No changes on MBP
Pazos et al., 2013	Piglets	Hypoxic ischaemia	1 mg/kg	i.v.	Post-HI	Pre-drug, at 30 and 90 min post-HI	No changes on MBP
Gonca and Darici, 2015	Rats	Myocardial infarction	50 μg/kg	i.v.	Pre-ischaemia	Pre-ishaemia At 1, 5 and 11 min post-ischaemia	No changes on BP or HR
Feng et al., 2015	Rabbits	Myocardial infarction	100 μg/kg	i.v.	Pre-reperfusion	At 15, 30 and 45 min post-drug	↑ BF
Garberg et al., 2016	Piglets	Hypoxic ischaemia	1 mg/kg	i.v.	Post-HI	Immediately post HI and at 30, 210 and 570 min post-HI	No changes on BP or HR

*ACSF, Artificial cerebrospinal fluid; BP, blood pressure; BNST, bed nucleus of the stria terminalis; CBD, Cannabidiol; HI, Hypoxic ischemia; HR, heart rate; h, hours; i.p., Intraperitoneal; i.v., intravenous; MBP, mean blood pressure; MCAO, middle cerebral artery occlusion; min, minutes; SBP, systolic blood pressure*.

### Blood pressure and heart rate

**Figure 2:**
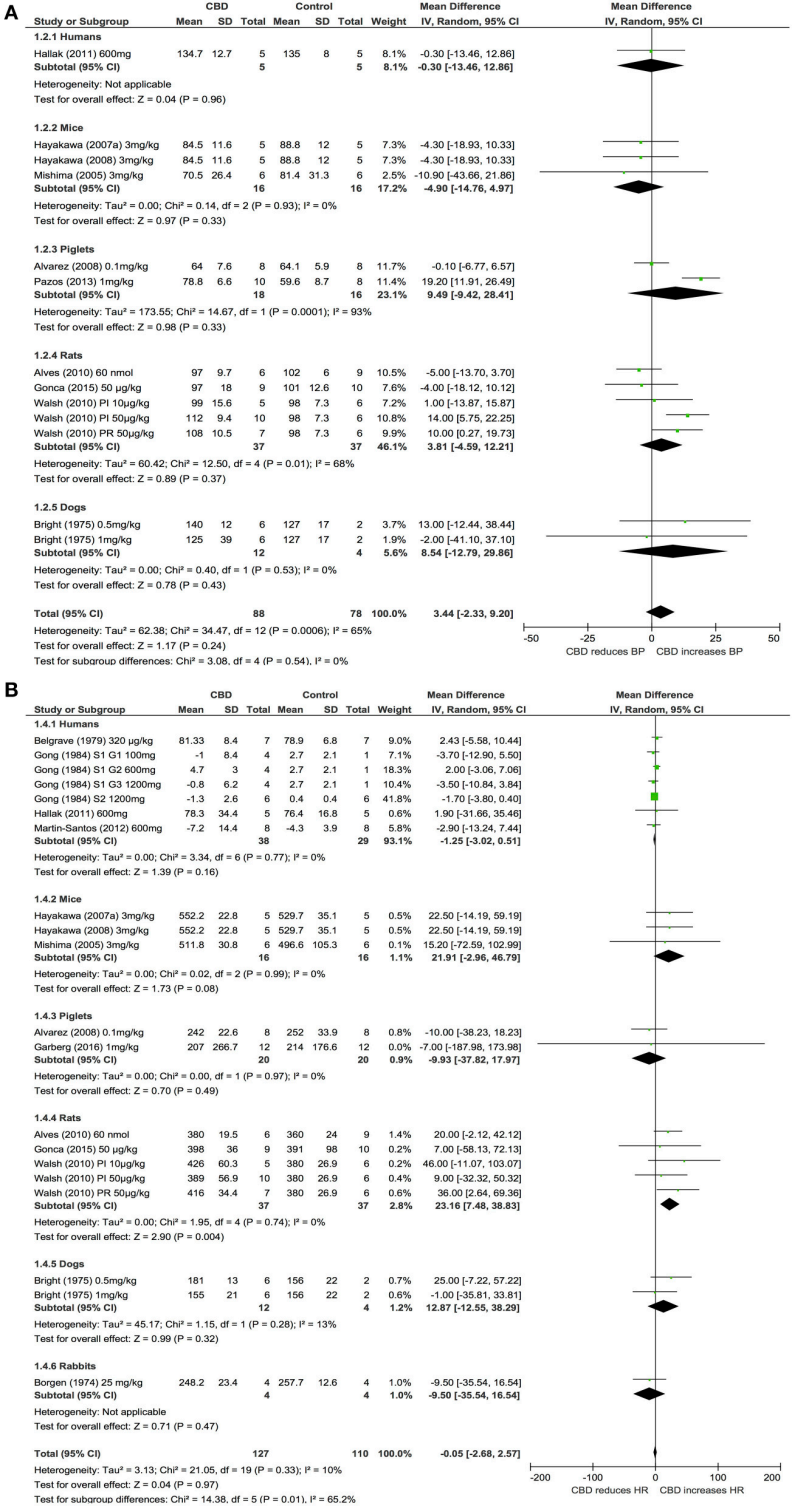
**Changes in BP (A)** and HR **(B)** induced by acute CBD dosing.

**Figure 3:**
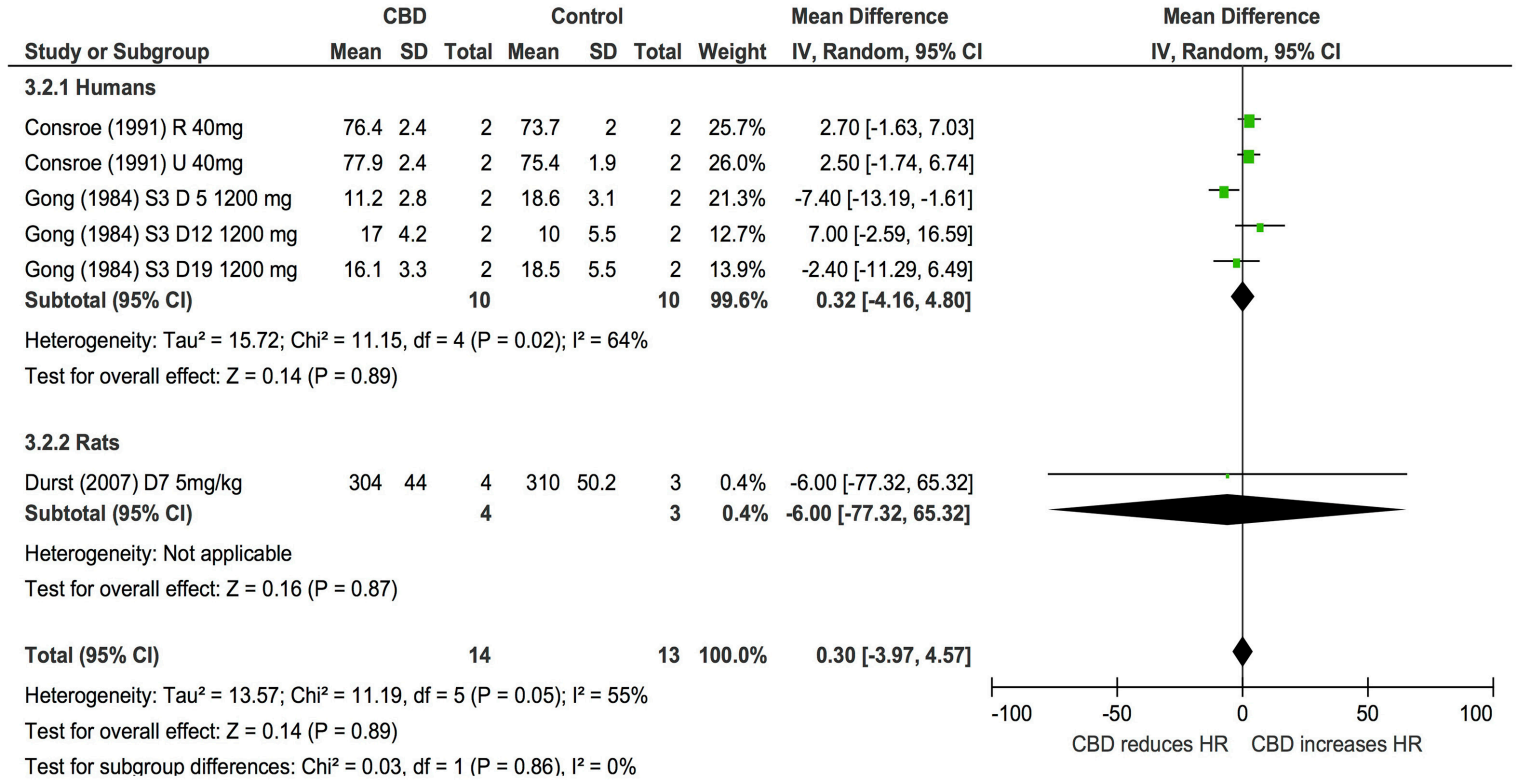
**Changes in HR induced by chronic CBD dosing**.

Ten publications assessed the acute effect of CBD administration on BP, and 15 publications assessed the effect of CBD administration on HR in 5 species, including humans, mice, dogs, rats, piglets, and rabbits (*n* = 403). Chronic dosing was assessed in 3 publications in 2 species, including humans and rats (BP: one study, *n* = 6; HR: 3 studies, *n* = 27). CBD had no effect on BP or HR after acute dosing (BP, MD 3, 95% CI −1.81, 7.8, *p* = 0.22; HR, MD −0.05, 95% CI-2.68, 2.57, *p* = 0.97, Figures 2A,B) or chronic dosing (HR MD 0.3, 95% CI −3.97, 4.57, *p* = 0.89, Figure 3). Within species analysis revealed that acute CBD dosing significantly increased HR in rats (*p* = 0.004, Figure 2B). Heterogeneity was statistically significant for BP measurements after acute CBD dosing (*p* = 0.0006; *I*^2^ = 65%) and HR measurements after chronic CBD dosing (*p* = 0.05; *I*^2^ = 55%; Figures 2, 3).

**Figure 4:**
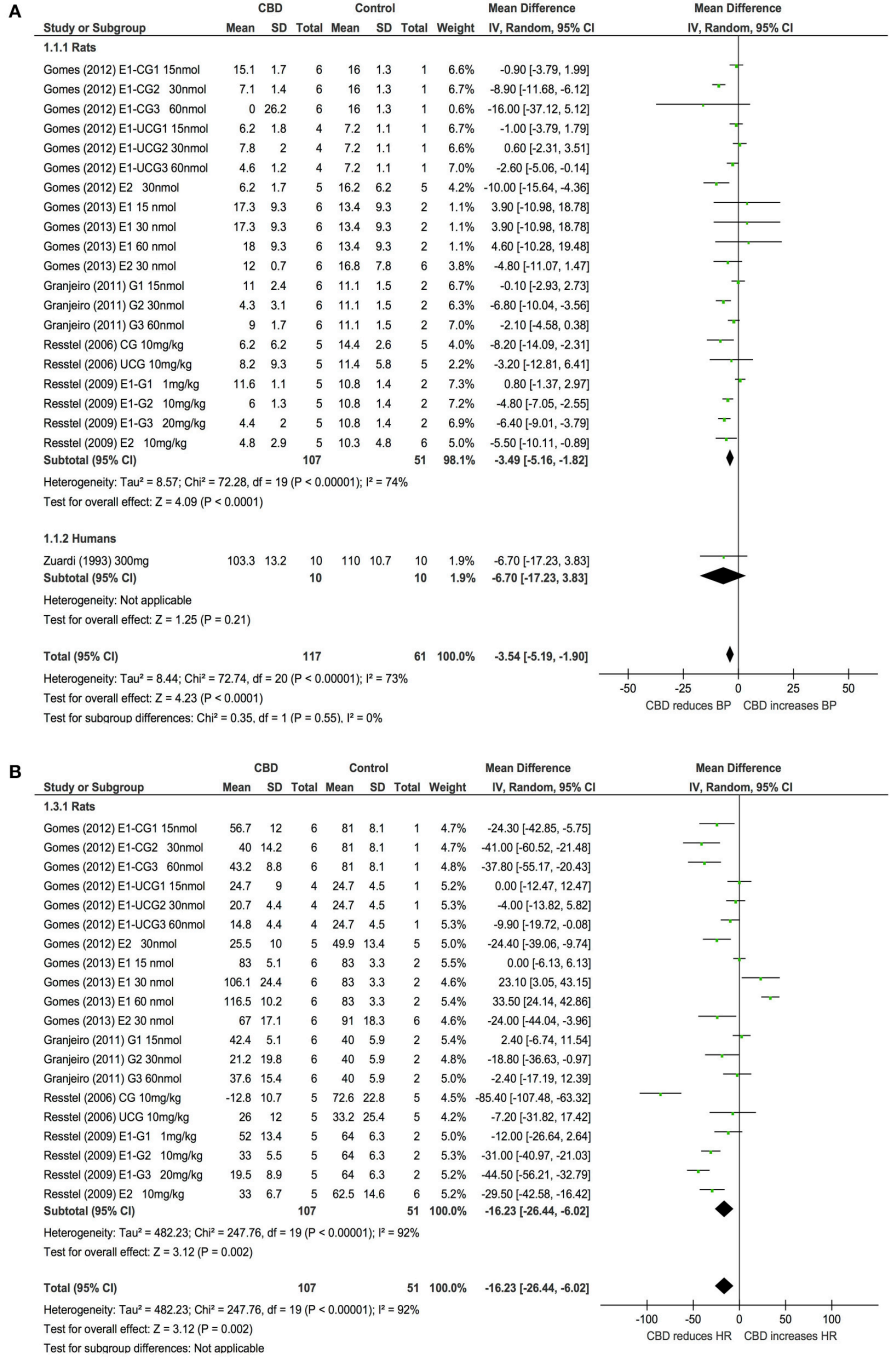
**Changes in BP (A)** and HR **(B)** induced by acute CBD dosing under stressful conditions.

Six publications assessed the effect of CBD administration on BP and HR in models of stress in rats and humans (*n* = 336). Overall, CBD administration significantly reduced the increase in BP (BP, MD: −3.54, 95% CI −5.19, −1.9, *p* < 0.0001, Figure 4A) and HR (HR, MD: −16.23, 95% CI −26.44, −6.02, *p* = 0.002, Figure 4B) induced by stress. Heterogeneity was statistically significant in both BP (*p* < 0.00001; *I*^2^ = 73%) and HR (*p* < 0.00001; *I*^2^ = 92%; Figure 4).

### Blood flow

**Figure 5:**
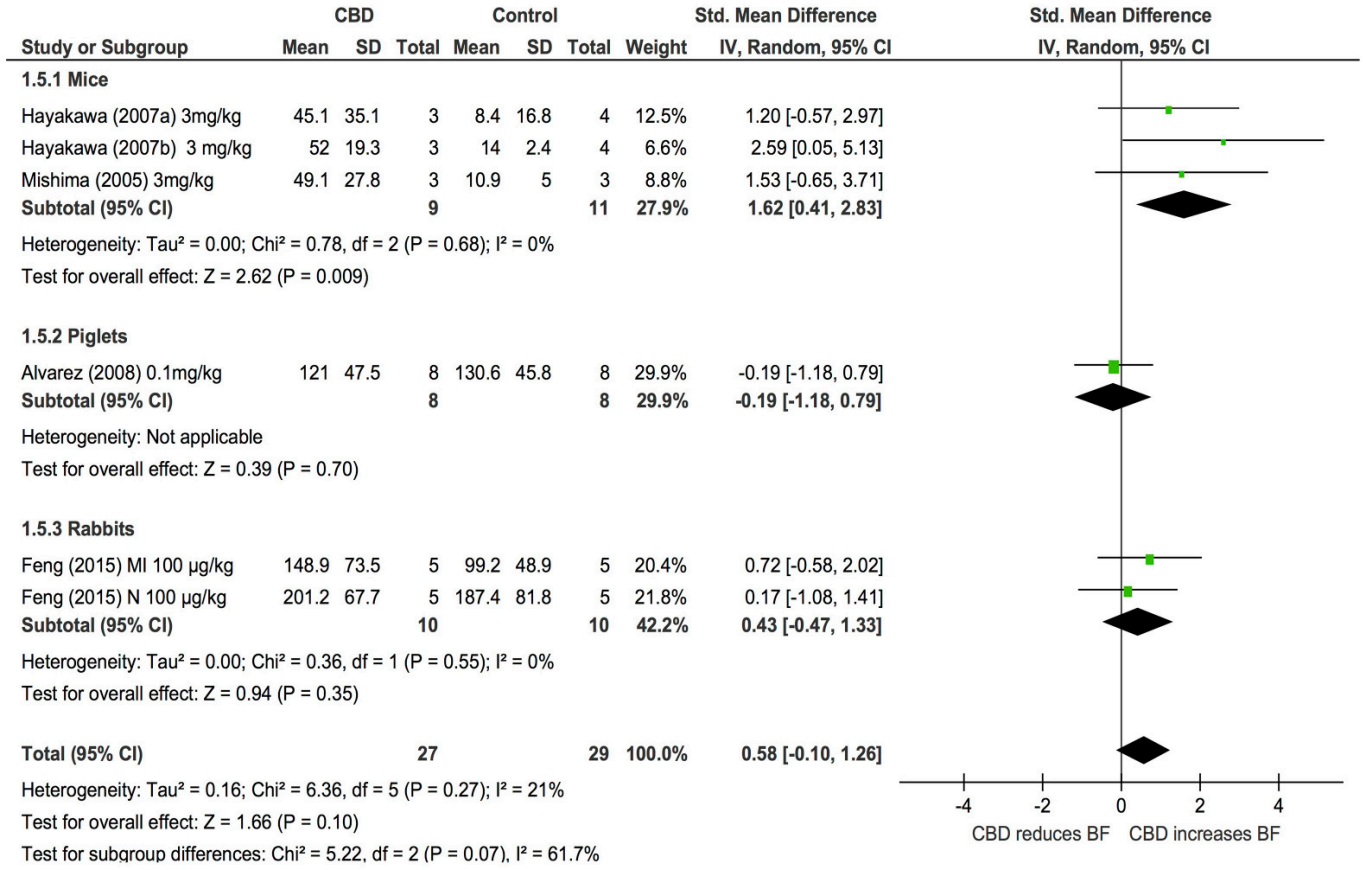
**Changes in regional blood flow induced by acute CBD dosing**.

Changes in BF after acute CBD dosing were assessed in 5 publications examining the effects of CBD in models of stroke or myocardial infarction in 3 species (mice, piglets and rabbits, *n* = 56). Overall, CBD had no effects on BF (SMD: 0.58, 95% CI −0.1, 1.26, *p* = 0.1). However, subgroup analysis showed that CBD significantly increased cerebral blood flow (CBF) in mouse models of stroke (*p* = 0.009, Figure 5); heterogeneity was not statistically significant (*p* = 0.27; *I*^2^ = 21%). As only one study assessed BF after chronic dosing, a meta-analysis was not applicable.

### Route of administration

We compared differences between local and systemic administration of CBD on haemodynamics. Local (intracisternal or intracerebral) administration of CBD was only used in studies on rats (4 out of 9 studies; 1 under control conditions and 3 under stressful situations). After systemic administration, there was a significant reduction in HR (*p* < 0.0001; 2 studies), but not after local (intracisternal or intracerebral) administration of CBD (*p* = 0.11; 3 studies).

### CBD dose-response on haemodynamics

**Figure 6:**
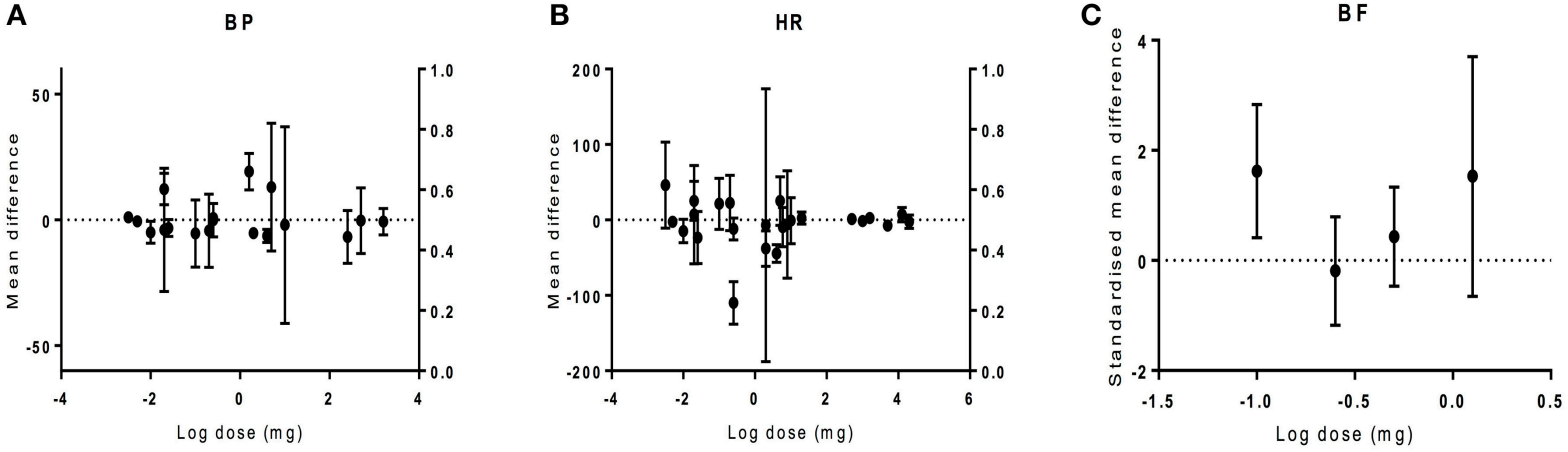
**The effect of CBD dose on haemodynamic responses *in vivo***. The mean difference (MD) in blood pressure (BP, **A**) or heart rate (HR, **B**), and standardized mean difference (SMD) in blood flow (BF, **C**) is plotted against the log dose (mg) for each study. Error bars represent 95% confidence intervals (CI).

The dose-response to CBD was analyzed to establish if there is a relationship between CBD dose and effect size. Doses ranging from 0.003 to 22800 mg were used in different species of different models. Overall, there was no relationship between drug dose and the size of the effect on haemodynamics (BP *p* = 0.81, HR *p* = 0.97, BF *p* = 0.97; Figure 6).

### Quality

Among the 25 included publications, 9 publications used randomisation and allocation concealment in their design, 6 reported blinding assessment of outcome and blinding of outcome measurements, 20 publications assessed more than one outcome, 12 conducted dose-response relationships, 19 assessed a time window for intervention, 4 measured outcomes >24 h post-drug and 2 publications provided incomplete data due to subject withdrawal There was no significant relationship between quality score and any outcome except in BF (Spearman's rho coefficient of BP, −0.1, *p* = 0.54, HR, 0.06, *p* = 0.66 and BF, 0.87, *p* = 0.01).

### Publication bias

**Figure 7:**
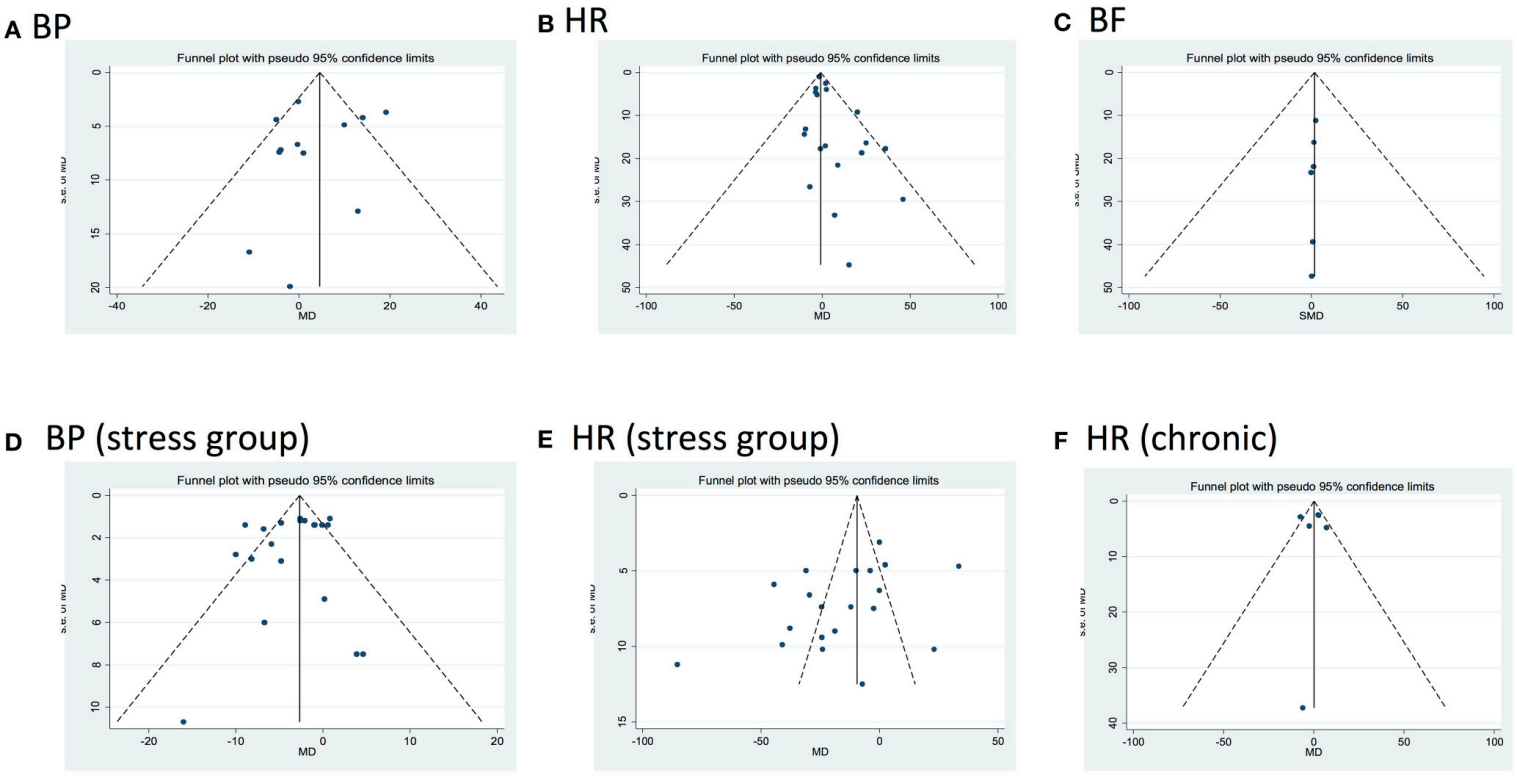
**Funnel plots for each outcome evaluating publication bias**. Standard error (SE) of the mean difference (MD) in blood pressure (BP) and heart rate (HR), or of the standardized mean difference in blood flow (BF, MD, or SMD, y axes) for each study is plotted against its effect size (horizontal axes). There was significant bias in HR after acute dosing (B, HR control *p* = 0.001; E, HR stress *p* = 0.049).

Egger‘s test showed no bias present except in studies assessing for changes in HR in either control or stressful conditions after acute administration of CBD (HR control *p* = 0.01; HR stress *p* = 0.049; Figure 7).

## Discussion

The aim of this study was to determine whether CBD alters haemodynamics *in vivo*. Our analysis has shown that acute and chronic dosing of CBD had no effect on BP, HR, or BF under control conditions. However, in stressful situations, CBD reduces the increase in MBP and HR observed in rats. Subgroup analysis revealed that acute CBD administration increases HR in mice and rats, and increases CBF in mouse models of stroke. Our analysis has highlighted the limited amount of human research carried out to date, and suggests that further work is required to assess the haemodynamic and regional BF impact of acute and chronic CBD administration in healthy volunteers and patients.

Overall, our meta-analysis showed that acute and chronic dosing of CBD had no effect on BP, HR, or BF under control conditions. However, there was significant heterogeneity with regards to species and model, dose and route of administration, and method and time of endpoint measurement (see Table 1) which makes it difficult to compare studies. It is possible that species differences may play a role. For example, Bright et al. found a significant increase in HR and BP with CBD (0.5 and 1 mg/kg) in anesthetized dogs (Bright et al., 1974, 1975), however, no changes were seen with CBD (25 mg /kg) in the HR of anesthetized rabbits (Borgen and Davis, 1974). It is also worth noting that most of the human data reviewed did not show any significant effects of CBD while significant effects were observed in animal studies. The time of cardiovascular measurements is also very important. For example, in a study that examined the cardioprotective effect of CBD (50 μg/kg i.v.) in rats showed that BP was significantly reduced post-CBD treatment compared to control two and a half hours post-ischaemia (Walsh et al., 2010). However, another study assessing CBD at the same dose and route of administration on cardiac arrhythmia (Gonca and Darici, 2015) found no change in BP, although this was only measured 11 min post-ischaemia, thus any potential later changes in haemodynamics are not reported. A greater number of homogenous studies are required to assess the haemodynamic effects of CBD under control conditions.

Our systematic review has highlighted that there are a limited number of studies examining changes in regional BF with CBD, with studies to date only examining changes in cerebral or myocardial BF. From the limited studies available, our analysis showed there were no significant changes in BF overall post-CBD administration. However, in mice and piglet models of stroke, either intraperitoneal or intravenous administration of CBD (3 mg/kg or 0.1 mg/kg, respectively) significantly reduced the infarct volume and increased the CBF after acute and chronic dosing (Mishima et al., 2005; Hayakawa et al., 2007a,b; Alvarez et al., 2008). Similarly, in a rabbit model of myocardial infarction, CBD treatment of 100 μg/kg modestly reduced the size of myocardial ischaemic injury and increased myocardial BF (Feng et al., 2015). Together, this suggests that further investigation on the effects of CBD on regional BF, particularly in pathological situations, is warranted.

There was no relationship found between the dose of CBD and the effect size. However, in conscious monkeys, toxicology studies showed that very large doses of CBD (150–300 mg/kg) caused bradycardia, and CBD doses >200 mg/kg caused heart failure and death (Rosenkrantz et al., 1981). However, it is worth noting that this would be equivalent to a dose of 14,000 mg in a 70 kg human. Intracisternal administration of CBD had no effect on the increase in BP or HR induced by acute restraint stress in rats while systemic administration did (Granjeiro et al., 2011). Similarly, no effect was seen in BP after intracerebral CBD injection in rats (Gomes et al., 2013). This suggests that systemic administration of CBD is required to observe changes in haemodynamics.

Although CBD did not affect haemodynamics under control conditions, our analysis did reveal effects of CBD in pathological situations. For example, in piglet models of hypoxic injury, intravenous administration of CBD (0.1 mg/kg) maintained a stable BP after hypoxic injury compared to control animals where a reduction in BP was observed (Alvarez et al., 2008). Also, in rats conditioned to stress (i.e., restraint or fear), CBD reduced the increase in HR and MBP (Resstel et al., 2006, 2009; Gomes et al., 2012). However, in mouse models of stroke there was no significant change in MBP or HR post-CBD (Mishima et al., 2005; Hayakawa et al., 2007a, 2008). This suggests that CBD may regulate the haemodynamics when they are altered at times of stress or acute illness.

Most of the identified relevant studies were pre-clinical, and data concerning the effects of CBD on haemodynamics in humans is limited (*n* = 36 for BP, *n* = 87 for HR). A single oral dose of CBD (320 μg/kg, 1 mg/kg, 100, 300, 600 or 1200 mg) had no effect on BP or HR in healthy volunteers under control or stressful situations (Belgrave et al., 1979; Zuardi et al., 1982, 1993; Gong et al., 1984; Borgwardt et al., 2008; Bhattacharyya et al., 2009, 2010; Fusar-Poli et al., 2009; Bergamaschi et al., 2011; Hallak et al., 2011; Winton-Brown et al., 2011; Martin-Santos et al., 2012). Likewise, after repeated CBD dosing of 1200 mg or 3 mg/kg for 20 or 30 days, respectively there were no apparent effects on HR, BP, or ECG compared with other treatment groups (Cunha et al., 1980; Gong et al., 1984). In clinical trials, chronic administration of CBD 300 mg, 10 mg/kg or 800 mg for 4½ months, 6 or 4 weeks, respectively, incurred no changes on the ECG, BP, or HR in patients of epilepsy, Huntington or schizophrenia disorders (Cunha et al., 1980; Consroe et al., 1991; Leweke et al., 2012). However, repeated oral dosing of CBD increasing from 100 to 600 mg/day over 6 weeks induced a reduction in standing BP by 10–20 mmHg in patients with dystonic movement disorders (Consroe et al., 1986). Conversely, a single dose of CBD (40 mg) given to patients with intraocular pressure increased systolic BP at 60 and 90 min post-sublingual administration (Tomida et al., 2006). Two studies of healthy volunteers and patients with social anxiety disorders showed CBD (400 mg) increased cerebral BF on the left parahippocampal and right posterior cingulate gyrus, respectively, but not in other brain regions when compared to control (Crippa et al., 2004, 2011). Bhattacharyya et al. (2010) suggested that the CBD effects on regional brain function during multi-tasking may be related to its effects on CBF (Bhattacharyya et al., 2010). Conversely, Borgwardt et al. (2008) suggested that the neural effects observed after CBD administration are unlikely to be a consequence of vascular effects, including CBF (Borgwardt et al., 2008). Together, this data would suggest that there are limited haemodynamic effects of CBD in humans, although further studies where this is the primary endpoint are warranted based on pre-clinical data reviewed in the present study.

### Limitations

There are several factors that limit the interpretation of the results of these studies and the understanding of the CBD effects on haemodynamics. In general, the primary aim of the studies reviewed was not to assess the haemodynamic effects of CBD. Some studies did not include an impartial measurement of BP or HR which may lead to bias in their outcome, or did not state the method of measurement. Due to the presence of heterogeneity in publications, outcomes after acute and chronic dosing should be interpreted with caution. After acute dosing, changes in haemodynamics at 2 h or the closest time point available to 2 h post-drug were used for analysis, however, depending on the route of administration, the peak changes in plasma CBD and therefore associated cardiovascular changes, will be different. In chronic studies, the length of drug administration also varied. For the analysis of the relationship between drug dose and effect size, the total dose up to the time point in which the haemodynamic was measured was used in the analysis, this also may affect review conclusions. Only 9 out of 24 publications used randomisation and 6 reported blinding assessment of outcome, parameters that should impact on study quality. However, we found no relationship between quality and effect size. Finally, in some publications involving comparison of several doses, the number of animals per control group was divided into the number of comparison groups to avoid re-counting the same animal more than once, thus resulting in smaller sample sizes and broader estimates of the variance.

## Conclusion

This meta-analysis and systematic review has highlighted the haemodynamic effects of CBD administration *in vivo*. The positive effects induced by CBD include maintaining the fall in BP after global hypoxia, reducing the increase in MBP and HR post-stress, and increasing BF in ischaemia-reperfusion models. It is possible that beneficial effects of CBD on haemodynamics occurs when the cardiovascular system is abnormally altered, suggesting that CBD may be used as a treatment for various cardiovascular disorders, such as hypertension, myocardial infarction and stroke. However, the findings from the reviewed studies were predominately preclinical and significant effects were only observed in animals. Data from human studies investigating the effects of CBD on haemodynamics is still very limited and we suggest that further research in humans under pathological conditions is required.

## Author contributions

SO and TE: Substantial contributions to the conception or design of the work. All authors: The analysis and interpretation of data for the work; Drafting the work or revising it critically for important intellectual content; Final approval of the version to be published; Agreement to be accountable for all aspects of the work in ensuring that questions related to the accuracy or integrity of any part of the work are appropriately investigated and resolved.

### Conflict of interest statement

The authors declare that the research was conducted in the absence of any commercial or financial relationships that could be construed as a potential conflict of interest.
